# The Role of Zr on Monoclinic and Orthorhombic Hf_x_Zr_y_O_2_ Systems: A First-Principles Study

**DOI:** 10.3390/ma15124175

**Published:** 2022-06-13

**Authors:** Eleonora Pavoni, Elaheh Mohebbi, Pierluigi Stipa, Davide Mencarelli, Luca Pierantoni, Emiliano Laudadio

**Affiliations:** 1 Department of Materials, Environmental Sciences and Urban Planning, Marche Polytechnic University, 60131 Ancona, Italy; e.pavoni@staff.univpm.it (E.P.); e.mohebbi@staff.univpm.it (E.M.); p.stipa@staff.univpm.it (P.S.); 2 Information Engineering Department, Marche Polytechnic University, 60131 Ancona, Italy; d.mencarelli@staff.univpm.it (D.M.); l.pierantoni@staff.univpm.it (L.P.)

**Keywords:** HfO_2_, Zr doping, Hubbard, optical properties, oxygen vacancies

## Abstract

HfO_2_ shows different polymorphs, including monoclinic and orthorhombic ones, that exhibit singular properties. Moreover, the character of HfO_2_ is also influenced by the Zr atoms as a doping agent. Here, an extensive study of the monoclinic *P2_1_/c* and the orthorhombic *Pca2_1_* polymorphs of HfO_2_, Hf_0.75_Zr_0.25_O_2_, and Hf_0.5_Zr_0.5_O_2_ is reported. For all six systems, density functional theory (DFT) methods based on generalized gradient approximations (GGAs) were first used; then the GGA + U method was settled and calibrated to describe the electrical and optical properties of polymorphs and the responses to the oxygen vacancies. Zr had different effects in relation to the polymorph; moreover, the amount of Zr led to important differences in the optical properties of the *Pca2_1_* polymorph. Finally, oxygen vacancies were investigated, showing an important modulation of the properties of Hf_x_Zr_y_O_2_ nanostructures. The combined GGA and GGA + U methods adopted in this work generate a reasonable prediction of the physicochemical properties of o- and m-Hf_x_Zr_y_O_2_, identifying the effects of doping phenomena.

## 1. Introduction

The discovery of ferroelectricity in hafnium oxides (HfO_2_) thin films [1] has been of crucial importance, thanks to the possibility of overcoming many of the problems related to perovskites-based field-effects transistor (FET) technology. Since the introduction in a manufacturing process by Intel in 2007, HfO_2_ has been vastly used as a high-k material, displaying a full CMOS (complementary metal–oxide–semiconductor) compatibility. The ferroelectric behavior originates from the development of nanoscale thin films [2]. The nature of the ferroelectricity is most likely related to the formation of a non-centrosymmetric polar crystal phase that is stable under specific conditions. As already mentioned, the ferroelectricity behavior of HfO_2_ is similar to that of perovskites materials but with a few peculiarities: HfO_2_ has a relatively wide bandgap (5.3 eV), large band offset with Si (less parasitic leakage), large coercive field (1 MVcm^−1^), and low permittivity. Importantly, the absence of an interfacial dead layer in HfO_2_ makes this material a promising candidate in thin-film technology, in contrast to perovskites-based material. Moreover, the mid-range dielectric constant allows the switching at reasonable voltage, even though the field strength required to obtain the reversal polarization is higher if compared to perovskites [3,4,5].

When an external electric field is applied to a ferroelectric material, a permanent polarization and hysteresis are induced; such an effect was found for Si-doped [1], Al-doped [6], and Y-doped HfO_2_ films [7]. However, HfO_2_ has been continuously studied to improve ferroelectricity and to extend its applicability in the semiconductor industry. In this context, the doping of HfO_2_ with Zr atoms was effective in obtaining ferroelectricity, although neither the pure HfO_2_ nor the pure ZrO_2_ films exhibit the same singular properties. It is well-known that the monoclinic structure (space group *P2_1_/c*) of HfO_2_ is the most stable; however, the presence of Zr determines the formation of an orthorhombic phase (*Pca2_1_* non-centrosymmetric space groups) which is closely related to ferroelectricity [8].

**Figure 1:**
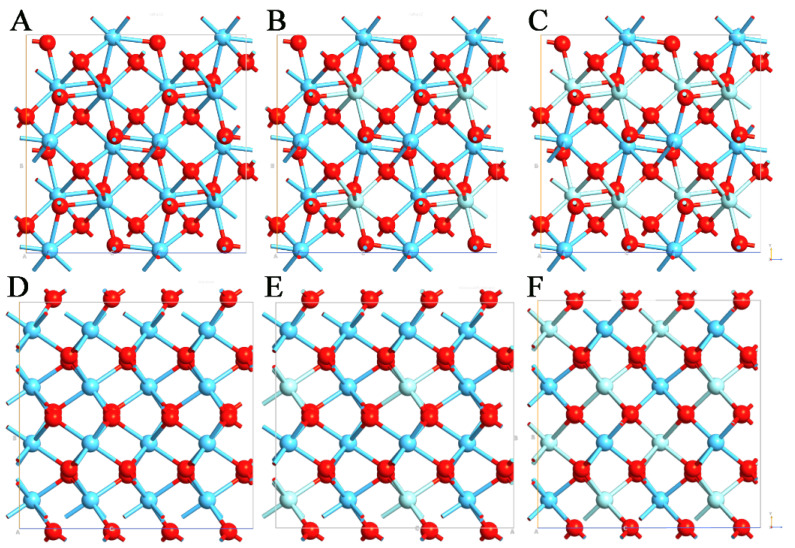
Structures of (**A**) m-HfO_2_, (**B**) m-Hf_0.75_Zr_0.25_O_2_, (**C**) m-Hf_0.50_Zr_0.50_O_2_, (**D**) o-HfO_2_, (**E**) o-Hf_0.75_Zr_0.25_O_2_, and (**F**) o-Hf_0.50_Zr_0.50_O_2_. Hf, Zr, and O are reported in blue, light blue, and red, respectively.

In the present work, we report an extensive theoretical study regarding the two mentioned polymorphs by doping different doses of Zr. Thus, six different systems were studied, the monoclinic and orthorhombic polymorphs of HfO_2_, Hf_0.75_Zr_0.25_O_2_, and Hf_0.5_Zr_0.5_O_2_ (Figure 1).

A wide comparison of different properties was performed by using the density functional theory (DFT) approach based on generalized gradient approximation (GGA) exchange-correlation functional [9]. Even if the DFT approach is known to be reliable for ground-state properties’ predictions, different studies indicate that conventional exchange-correlation (xc) functionals often underestimate the bandgap of semiconductors concerning the experimental studies [10]. To overcome this problem, a computational strategy that is able to include the correlation effect with reasonable computational effort is the GGA + U approach. Using this method, the correlation effect is incorporated through the on-site Coulomb interaction Hubbard (U) value that needs to be calibrated. The inclusion of corrective terms confers a qualitative improvement in comparison to the GGA based DFT approach for excited-state properties (such as DOS and optical properties [11]).

The on-site U for Hf 5^d^, Zr 4^d^, and O 2^p^ electrons was considered even if the U correction is typically only applied on the d and f orbitals of metal entities. The inclusion of 2p electrons of O entities in the U treatment allows us to better describe these strong Coulomb interactions, making the U^p^ values of O to be crucial in metal oxides [12,13]. Hf U^d^, Zr U^d^, and O U^p^ values were settled by using on-site repulsion of 6, 5.8, and 4 eV, respectively, fitting these values to the DFT approach adopted.

For all of these reasons, the combined employment of DFT and DFT + U functional was adopted to give the most reasonable energy stability and structural parameters of the systems. In detail, the effects of Zr doping on lattice parameters were elucidated by using the DFT-GGA approach, and then the electrical and the optical properties of all six models were calculated by adopting the DFT-GGA + U method.

It is well-established that oxide defects adversely affect the functionality and reliability of a wide range of semiconductor devices strongly affecting the physical–chemical properties of the material and thus decisively impact the performances of the respective devices. With this aim, the defects’ formation was also considered with the DFT + U approach to identify the effects of oxygen vacancies on the structural and electrical properties of HfO_2_ and Hf_x_Zr_y_O_2_ polymorphs. Since the *PCa2_1_* phase and the Zr amount are correlated to ferroelectricity, differences in the defects’ propensities are expected between polymorphs.

## 2. Materials and Methods

A quantum ATK atomic-scale modeling platform was used to model all polymorphs and to perform all calculations [14]. Six structures, namely three monoclinic (*P2_1_/c*) and three orthorhombic (*Pca2_1_*) polymorphs, were modeled: (i) HfO_2_ with 0% of Zr substitution, (ii) Hf_0.75_Zr_0.25_O_2_ with 25% of Zr substitution, and (iii) Hf_0.5_Zr_0.5_O_2_ with 50% of Zr substitution with respect to the total Hf amount. To discover the effects of different amounts of Zr in each polymorph, the Hf ions were randomly substituted, and all six models were simulated. The electron basis was expanded in linear combination, using the atomic orbital (LCAO) method for Hf, Zr, and O entities, resembling the SIESTA formalism [15]. All simulations were carried out by using the Perdew–Burke–Ernzerhof (PBE) GGA density functional for the electron xc energy [16]. For each atom, the ionic cores are represented by norm-conserving (NC) PseudoDojo (PDj) pseudopotentials [17]. The 5d^2^ and 6s^2^ electrons of Hf and 4d^2^ and 5s^2^ electrons for Zr are explicitly treated as valence, while the 2s^2^ and 2p^4^ electrons are the obvious valence electrons for O atoms. Correct on-site repulsion values were calibrated, and then the Hubbard (U) values of 6, 5.8, and 4 eV for Hf^d^, Zr^d^, and O^p^ valence electrons were chosen, respectively, to reproduce the bandgap values of HfZrO_2_. Since the description of charge density in the core region could overlap valence density when U values are included, the choice of the correct NC PDj is greatly important because the charge density of the electron core was reproduced within a short cutoff radius, thus avoiding an underestimation of the xc energy. To model the polymorphs, the periodic boundary conditions (PBCs) were used along all axes; in this way, it is possible to avoid problems with boundary effects caused by the finite size and to reduce the calculation time while maintaining high accuracy. The energy cutoff was fixed at 1200 eV, and the Brillouin-zone integration was performed over a 15 × 15 × 15 k-points grid for the modeled *P21/c* and Pca21 polymorphs. These parameters assure the total energy convergence of 5.0 × 10^−6^ eV/atom, the maximum stress of 2.0 × 10^−2^ GPa, and the maximum displacement of 5.0 × 10^−4^ Å. The polarization of o-*Pca2_1_* polymorph was obtained by the modern theory of polarization [18] and the Berry phase operator method. The total polarization is assumed to be the sum of the electronic and ionic contributions. The first one (*P_i_*) is calculated by using a simple classical electrostatic sum of point charges, as shown in Equation (1):

(1) $$P_{i}=\frac{\left|e\right|}{\Omega }\underset{\nu }{\sum }Z_{ion}^{v}r^{v}$$

where *Ω* is the unit cell volume, *Z^v^_ion_* is the valence charge, and *r^ν^* is the position vector of the *ν* atom.

The second contribution (*P_e_*) to the polarization is obtained as Equation (2):

(2) $$P_{e}=-\frac{2\left|e\right|i}{{\left(2\pi \right)}^{3}}\int_{A}dk_{⊥}\overset{M}{\underset{n=1}{\sum }}\int_{0^{U_{k},n\left|\frac{\partial }{\partial k}\right|u,<,n}}^{G}dk$$

where the sum runs over occupied bands, and *k* is parallel to the direction of polarization. The *G* term is a reciprocal lattice vector in the same direction. The states *U_k,n_* > are the cell-periodic parts of the Bloch functions, *ψ_k,n_ (r)* = *u _k,n_ (r) e ^ikr^*. The last integral is known as the Berry phase [19]. Oxygen vacancies were explicitly treated for each unit cell by putting two O atoms in a ghost configuration. This approach allows for the maintenance of the basic functions associated with the atoms selected when removing them to create the vacancies. The energy formation of a defect *X* in charge state *q* is defined as Equation (3):

(3) $$E^{f}=\left[X^{q}]=E_{tot}[X^{q}]-E_{tot}[HfZrO_{2}\right]-\sum n_{i}\mu_{i\;}+qE^{f}+E_{corr}$$

where *E_tot_ [X^q^]* is the total energy derived from a supercell calculation containing the defect *X*, and *E_tot_ [HfZrO_2_]* is the total energy for the perfect crystal using an equivalent supercell. The integer *n_i_* indicates the number of atoms of type *i* (host atoms or impurity atoms) that were added to (*n_i_* > 0) or removed from (*n_i_* < 0) the supercell to form the defect, and the *μ_i_* corresponds to the chemical potentials of these species. Chemical potentials represent the energy of the reservoirs with which atoms are being exchanged. The analog of the chemical potential for “charge” is given by the chemical potential of the electrons, i.e., the Fermi energy *E^F^*. Finally, *E_corr_* is a correction term that accounts for finite k-point sampling in the case of shallow impurities or elastic and/or electrostatic interactions between supercells.

## 3. Results and Discussions

As previously reported, the GGA/PBE and GGA/PBE + U methods were used in combination to calculate the different properties of six systems. The results are organized as follows: The geometry optimizations, lattice parameters, ground state cohesive energy formation, and tensile stress values were calculated by using the DFT approach and are reported in the first part. Then the second section is dedicated to the fitting of the U values to further use. Next, the electrical and optical properties of the six optimized systems are investigated by using the GGA + U^d^ + U^p^ approach, and the respective results are reported in the third section. In the fourth section, the same approach is used to model oxygen vacancies, calculate the defect formation energy, and compute bandgap structure variations for each system.

### 3.1. Effects of Zr on Lattice Parameters

HfO_2_ and Hf_x_Zr_y_O_2_ are characterized by different polymorphs, the monoclinic (m−) and orthorhombic (o−) ones, with space groups of *P2_1_/c* and *Pca2_1_*, respectively (Figure 1). Each of the two polymorphs was used to evaluate the effect of the presence of Zr. Table 1 reports the values of the calculated lattice parameters for the monoclinic and orthorhombic configurations of (i) HfO_2_ with 0% of Zr substitution, (ii) Hf_0.75_Zr_0.25_O_2_, and (iii) Hf_0.5_Zr_0.5_O_2_ with 25% and 50% of Zr substitution to the total amount of Hf ions, respectively.

Lattice energy minimized for m-HfO_2_ was obtained by the optimization of the atomic positions and systematically altering the size and angle of the unit cell. After optimization of the lattices, unit cell dimensions of 5.068, 5.135, and 5.292 Å were found for a, b, and c, respectively. The lattice vectors slightly decreased by imposing Zr in the system.

The *Pca2_1_* polymorph is directly correlated to the ferroelectricity [22], which is due to the formation of the non-centrosymmetric polar orthorhombic phase. The displacement of four polarization-related oxygen atoms per unit cell leads to the polarization of the phase. The optimized lattices for o-HfO_2_ were 5.231, 5.008, and 5.052 Å for a, b, and c vectors, respectively; this is in line with other results [23].

The effect of Zr on the *Pca2_1_* unit cell is generally more evident than that observed for the *P2_1_/c* polymorph, and, in particular, a sensitive decrease in the c vector was detected in o-Hf_0.5_Zr_0.5_O_2_. Since the c vector follows the direction of spontaneous polarization of the phase, these data suggest that Zr has a significant role to modulate the ferroelectric property of this polymorph.

**Figure 2:**
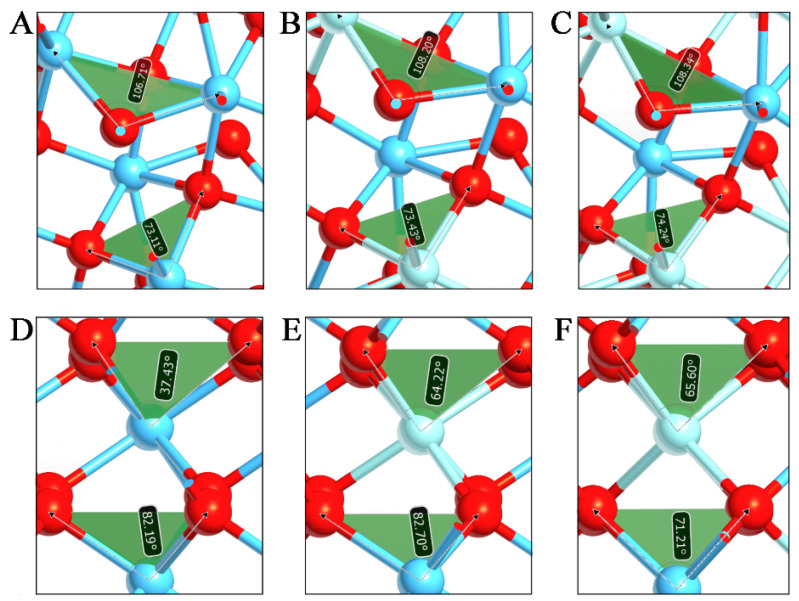
Bond angles of (**A**) m-HfO_2_, (**B**) m-Hf_0.75_Zr_0.25_ O_2_, (**C**) m-Hf_0.5_Zr_0.5_O_2_, (**D**) o-HfO_2_, (**E**) o-Hf_0.75_Zr_0.25_ O_2_, and (**F**) o-Hf_0.5_Zr_0.5_O_2_ systems. Hf, Zr, and O are reported in blue, light blue, and red, respectively.

In Figure 2, the m-phases of HfO_2_, Hf_0.75_Zr_0.25_O_2_, and Hf_0.5_Zr_0.5_O_2_ systems are reported, whose bond angles undergo only moderate changes when Zr replaces Hf atoms. Inside the unit cell, the angle Hf-O-Hf (106.71°) slightly increased in Zr-O-Hf (108.20° and 108.34° for 25% and 50% of Zr substitution, respectively). The same trend, but less pronounced, is observed for the O-Hf-O angle (73.11°), which increased in O-Zr-O (73.43° and 74.24° for 25% and 50% of Zr substitution, respectively). This assumption is in line with the negligible differences found for the cell vectors as the amount of Zr increases. More substantial effects can be observed for the o-polymorphs, where the Zr intercalation has a high impact on the bond angles with the asymmetric O entities (Figure 2). This influence is directly linked to the Zr amount in the Hf_0.5_Zr_0.5_O_2_, and the results show an important rearrangement of atom coordinates along the c vector. Based on this evidence, the replacement of 50% of Hf with Zr atoms has the peculiar role of stabilizing the o-polymorph while preserving the necessary asymmetry for the ferroelectric behavior.

**Figure 3:**
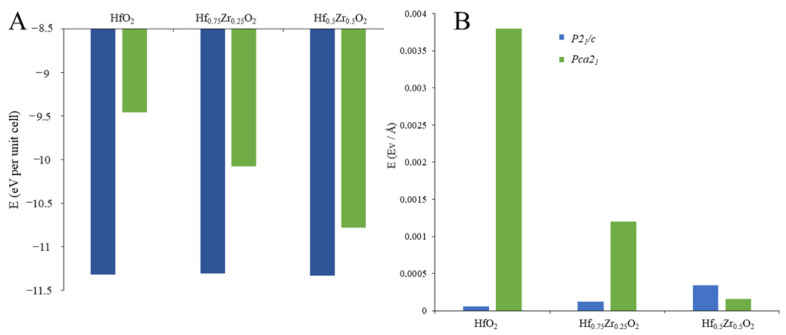
Ground state cohesive energy formation (**A**) and tensile stress values (**B**) of monoclinic (blue) and orthorhombic (green) HfO_2_, Hf_0.75_Zr_0.25_O_2_, and Hf_0.5_Zr_0.5_O_2_.

In order to gain deeper insight into the behavior of the phases and to underline the role of Zr, the PBE–GGA approach was again used to compute the ground-state cohesive energy of the polymorphs (Figure 3A). The direct comparison between m- and o-HfO_2_ phases outlined higher stability for the m-polymorph, as already reported in Reference [24]; negligible energetic variations were found for *P2_1_/c* phases when Zr replaces with Hf atoms. Therefore, the zirconium ion exhibits an excellent doping agent for the hafnium-based oxides [9]. An opposite trend was observed for the orthorhombic polymorphs, in which the Zr doping drastically decreased the formation energy. This effect appeared to be Zr-content dependent; that is, the higher the Zr percentage, the lower the formation energy, confirming the high impact of zirconium on the phase properties and the stabilization of the ferroelectric crystal.

The stabilization effect of Zr atoms to the orthorhombic structure is also confirmed by internal stress values (Figure 3B), which quantify the internal constriction factors in the unit cell. The m-polymorphs do not show a variation of the internal stress after the Zr inclusion, as is predictable by the minor changes in the lattice parameters when moving from undoped HfO_2_ to Hf_0.75_Zr_0.25_O_2_ and Hf_0.75_Zr_0.25_O_2_. On the contrary, DFT calculations detected internal stress of unit cells for the o-HfO_2_ (0% of Zr); the stress value drastically decreases when zirconium is included, reaching 7.6 × 10^−4^ eV/Å at 50% of Hf replaced by Zr.

Therefore, the increment on Zr concentration seems to induce a structural transition and an internal stabilization for the orthorhombic polymorph. These results are consistent with experimental data indicating tensile stress on the ferroelectric films [25] and showing that a composition close to 50% Hf and 50% Zr tends to favor ferroelectricity [26].

### 3.2. DFT + U Calibration

**Figure 4:**
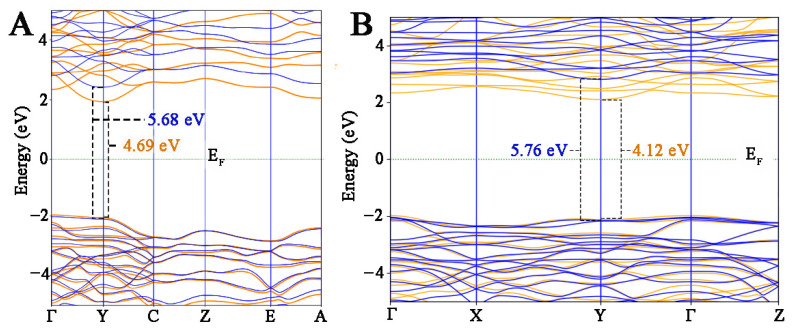
Calculated electronic band structure of m-HfZrO^2^ (**A**) and o-HfZrO_2_ (**B**), using GGA (orange) and GGA + U (blue) approach. Fermi level is highlighted by a dotted green line.

As previously described, the DFT approach is used to compute lattice parameters, cohesive energy formation, and internal stress values. Since the GGA–PBE method is not reliable to compute excited state properties as dielectric functions, the inclusion of corrective terms to the canonical DFT approach is necessary to obtain the partial density of states (PDOS) and bandgap variations induced by oxygen vacancies. Through the inclusion of on-site Coulomb interaction Hubbard (U) terms, it is possible to better describe the chemical bonds involving d and p electrons of Hf, Zr, and O atoms and to improve qualitatively the electronic descriptions. Zr U^d^, Hf U^d^, and O U^p^ values were settled to 5.8, 6, and 4 eV, respectively. To avoid non-physical states, the U values were calibrated by computing the already known bandgap values of m- and o-HfZrO_2_ polymorphs. Since the U values fitted under one set of technical assumptions could not be used for different DFT approaches, identifying the optimal values to use together with specific pseudopotentials represents a crucial step. It is useful to emphasize that there are no data reported in the literature about the use of the PseudoDojo library for the proposed HfZrO_2_ polymorph. Even if the coordinates and the structural parameter effects are different in the HfZrO_2_ polymorphs under study, the Hf^4+^, Zr^4+^, and O^2-^ entities do not change. This means that, since U depends on the projectors used, in our case, the same U values can be applied for more than one polymorph [27]. To be sure about the use of univocal U values for both polymorphs, the bandgap values calculated, including/excluding U values, were reported and compared to one another, and then the optimal values were obtained (Figure 4). Following the calibration of the U values, the effective Hubbard in GGA + U calculation tended to upper shift the minimum conduction band, providing a larger gap between the lowest conduction band and the highest valence band; this behavior was observed for both HfZrO_2_ polymorphs. With the previously reported U, the bandgap values obtained were 5.68 and 5.76 eV for the m- and o-phases, respectively; these results are in line with the experimental results of Reference [28]. These calculations confirmed the reliability of the fitted U values to compute the excited states’ properties for both of the polymorphs.

### 3.3. Electrical and Optical Properties Calculation

In the next step, DFT + U approximation was used to investigate the electrical and optical properties of m-*P2_1_/c* and o-*Pca2_1_* Hf_x_Zr_y_O_2_ polymorphs.

To determine the relative values of charges from wave functions, focusing on a qualitative point of view, the Mulliken population analysis [29] was performed (Table 2). For the *P2_1_/c* HfO_2_, the transferred charge from the Hf to O atoms is 0.840 atomic unit (*a.u*.). Following the doping, a proportional decrease in charge transferred into O is evident with 0.826 and 0.812 for 25% and 50% doped m-polymorphs, respectively. A similar trend was observed for *Pca2_1_* polymorphs when Zr was absent. Instead, when Zr was included in the polymorph, the charge transfer rose remarkably in O atoms, with 0.882 and 0.981 for 25% and 50% doped o-polymorphs, respectively. These results suggest that Zr incorporation into crystals leads to an increase in charge transfer from Hf and Zr into O atoms, and this effect is evident in o-phases, in particular, on Hf_0.5_Zr_0.5_O_2_.

**Figure 5:**
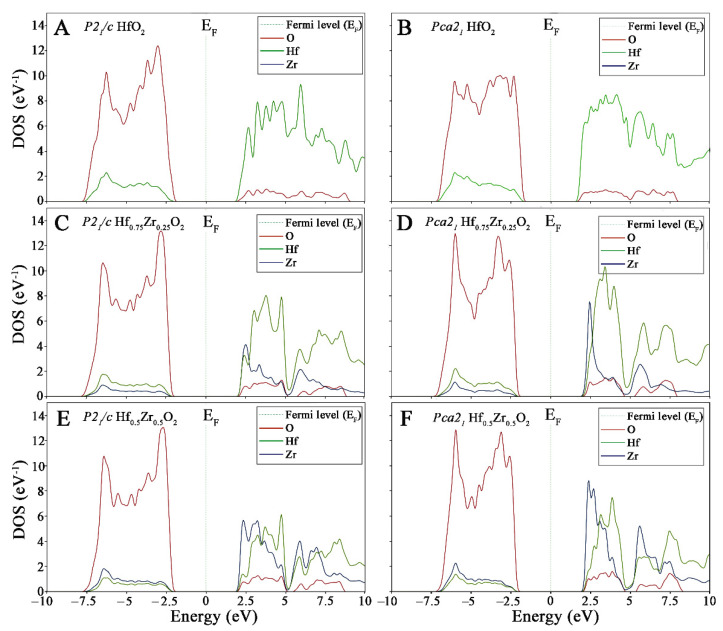
Partial density of state of HfO_2_ (**A**,**B**), Hf_0.75_Zr_0.25_O_2_ (**C**,**D**), and Hf_0.5_Zr_0.5_O_2_ (**E**,**F**). The m-*P2_1_/c* and o-*PCa2_1_* are reported to the left and right columns, respectively.

The GGA/PBE + U approach was also used to identify differences in bandgap values by computing the partial density of states (PDOS) and to compare the behavior of the different phases (Figure 5). For all systems, only one feature in the valence band was evidenced. Between an energy range of −7.5 and −2 eV, the main contribution of valence band maximum (VBM) originated from O 2^p^ orbitals. On the other hand, the energy ranges from 2.2 eV upward represent the conduction band maximum (CBM), which was mainly composed of Hf 5^d^ orbitals. Upon doping into the two pure systems by introducing dopant Zr atoms, the VBM is still mainly attributed to O 2^p^, while the CBM depends on Hf 5^d^ and Zr 4^d^ orbitals. More, with the inclusion of Zr, impure states are formed between the Fermi level and CBM (Figure 5E,F).

In general, the intensity of the PDOS peaks is sensitive to the geometrical structure around the Hf atoms. Obviously, as the Zr concentration increases, the CMB is divided into two parts from the energy range of around 5.00 eV. Our inspection of Figure 5C,D reveals that Hf^3d^ and Zr^3d^ orbitals have more contributions in PDOS plots, while O ^2p^ possesses less content. Furthermore, the Zr defect enhanced the peak intensity for *Pca2_1_* more than the *P2_1_/c* HfO_2_ polymorph. As Zr doping increased from 25% to 50% (Figure 5E,F), similar results appeared in the CMB state of both polymorphs by dropping peak intensities; however, the intensity of the Zr^3d^ peak in *P2_1_/c* HfO_2_ became weaker than one calculated for the *Pca2_1_* HfO_2_ structure, meaning that the former compound is more affected by the presence of Zr, while the latter structure undergoes slight changes.

One of the most interesting behaviors from PDOS is the change in the Zr contribution, moving from Hf_0.75_Zr_0.25_O_2_ to Hf_0.5_Zr_0.5_O_2_ in o-polymorph. The VBM and CBM energies were also chosen to estimate the bandgap values. The monoclinic structures are characterized by bandgap values of 5.24, 5.62, and 5.68 eV for 0%, 25%, and 50% of Zr doses. In the o-HfO_2_, a similar trend was observed that is relevant to the Hf and O contributions. The extrapolated bandgap is 5.63, 5.81, and 5.76, moving from 0%, 25%, and 50% of Zr contributions, respectively.

**Figure 6:**
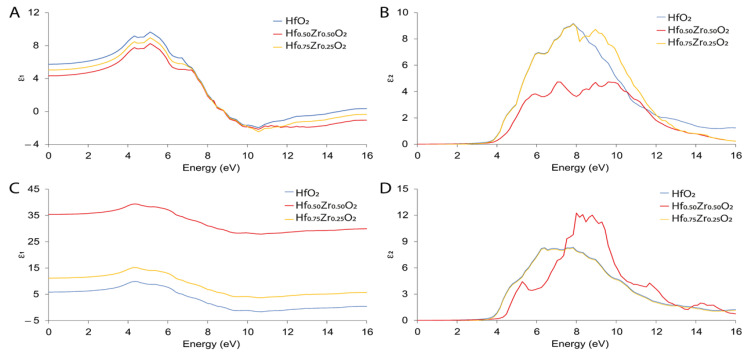
Dielectric function for m-phase (**A**,**B**) and o-phase bottom (**C**,**D**) of Hf_x_Zr_y_O_2_.

The effects of Zr on the real part (ε_1_) and imaginary part (ε_2_) of dielectric function for m- and o-HfZrO_2_ were calculated on a wide energy range (Figure 6). As reported, the ε_1_ value measured for m-polymorphs (Figure 6A) at 0 eV was 5.73, 5.02, and 4.33 for HfO_2_, Hf_0.75_Zr_0.25_O_2_, and Hf_0.5_Zr_0.5_O_2_, respectively. It is worth mentioning that the maximum exhibited constants are 8.2, 8.9, and 9.4, respectively, reaching the maximum static dielectric constant at a lower energy range. This indicates that these systems could be easily polarized. The peaks below zero reveal the metallic behavior of the targeted systems at an energy range of 8.4 to 15 eV, which is ascribed to the reflection of the electromagnetic radiation due to the anomalous dispersion approach. On the other hand, the ε_2_ was in the order of 10^−4^ for all m-phases at 0 eV, showing differences between them up to 4 eV (Figure 6B); this is in agreement with other studies [23].

The o-phase of Hf_x_Zr_y_O_2_ shows a different behavior due to its ferroelectric character that influences the response of the material to the applied field. In detail, the calculated ε_1_ has different values depending on the Zr amount, moving from 5.88 (without Zr) to 36.06 (in Hf_0.5_Zr_0.5_O_2_) at 0 eV (Figure 6C).

These values are higher compared to those of the m-structures and are in line with the experimental studies that remark the high values of dielectric constant for the ferroelectric phase [30]. The calculated ε_2_ remained in the order of 10^−3^ up to 3.36 eV (Figure 6D). The optical transition of the undoped o-polymorph situated in 6.1 eV can be attributed to the intrinsic transition between O 2^p^ states (at valence band) and Hf 5^d^ states (at conduction band). More probably, this change can be ascribed the incorporation of Zr and the shift in the transition to a higher energy range, from 6.1 to 8.2 eV, revealing the bandgap increase in doped polymorphs. To conclude, Zr does not have marked effects on the optical properties of m-phases, while it appeared mandatory to modulate the dielectric properties of the ferroelectric o-polymorphs.

### 3.4. Oxygen Vacancies Effects

To better describe the role of Zr on properties of different Hf_x_Zr_y_O_2_ phases, oxygen vacancies were included in the calculation, putting two O entities in ghost configuration. This approach consists of leaving the basis functions associated with an atom when removing it to create the vacancy. Since each unit cell is constructed of twelve atoms, and eight of them are oxygen entities, the explicit assumption of two considered vacancies leads to an important defect of materials. To quantify the effect of vacancies, the defect formation energy was computed for each polymorph. The calculation of defect formation energy includes (i) the vibrational (phonon) contributions because the creation of a defect modifies the chemical bonds and, thus, the bond strength; (ii) the electronic contributions, which are commonly small for semiconductors but can be sizable for metals; and (iii) the magnetic excitations. Defect-formation energies were identified (Table 3) by computing the total energy formation of each system with and without defects. The values obtained were always positive, and this is not a surprise because, in the case of negative values of the defect formation energy, the not-defected crystals would be unstable. Moreover, polymorphs exhibited different aptitudes to suffer defects, and Zr had an important role in modifying the tendency of different systems to undergo oxygen vacancies. The monoclinic phase always shows a major propensity to suffer oxygen vacancies, and the inclusion of Zr seemed to slightly decrease the formation energy of oxygen vacancies. The reason could be related to the different strength of chemical bonds of Zr with respect to Hf, since the first has a less energetic electronic level than the second. On the other hand, the orthorhombic phase was found to be less likely to undergo oxygen vacancies, and the reason could be ascribed to the ferroelectric behavior of this polymorph. The ferroelectricity, which was correctly reproduced by the simulations, could be at the basis of this energetic difference with the monoclinic phase since the oxygen entities that were placed in the ghost configuration were directly involved in the spontaneous polarization of the phase. Moreover, the inclusion of Zr strongly increased the energy of the oxygen vacancies’ formation, and this is probably due to the internal stress modulated by Zr, which finds the best values with 50% of metal.

**Figure 7:**
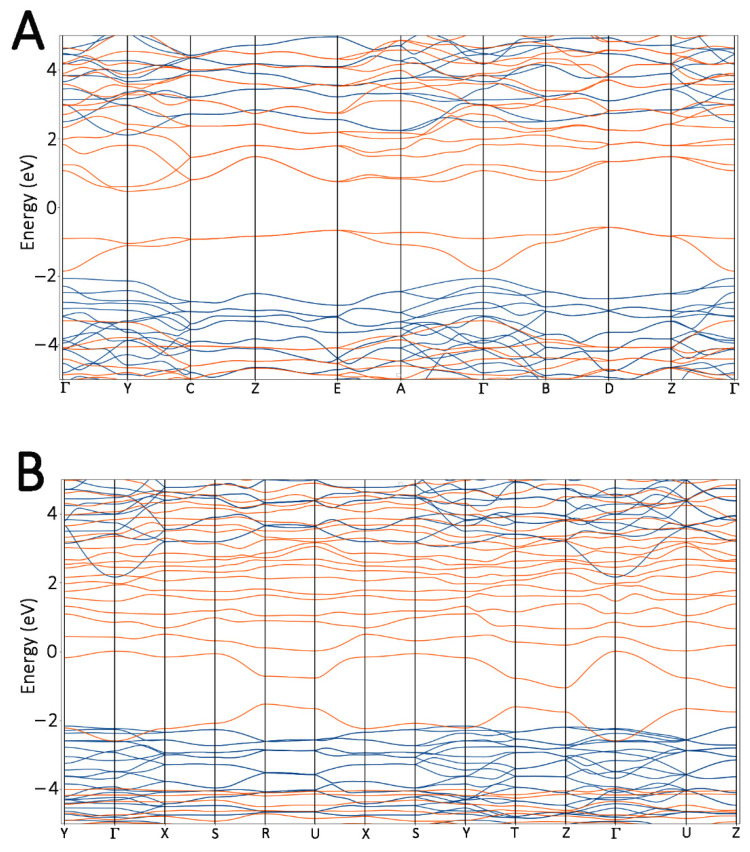
Bandgap structures of m- (**A**) and o- (**B**) Hf_0.5_Zr_0.5_O_2_. The blue lines highlight the bands of no-defect models, while orange lines outline the bands of defected systems.

This result is extremely important to identify the relative stability of the ferroelectric Hf_x_Zr_y_O_2_ polymorphs and to predict the best conditions in the vision of devices’ development. The effect of oxygen vacancies on the electrical properties of the polymorphs was also investigated. With the aim to remark upon the differences, the band structures with and without defects were calculated by using the previously described GGA/PBE + U approach. Since no differences were found when different amounts of Zr were included, only the results for Hf_0.5_Zr_0.5_O_2_ polymorphs (with and without vacancies) are reported (Figure 7).

An increase in the numbers of valence and conduction bands was found in the defective systems of both polymorphs, and the calculated bandgap was always lower than that computed for the pure Hf_x_Zr_y_O_2_ phases. Another evident effect of oxygen vacancies was that the bandgap underwent a shrinkage, and this is directly related to the delocalized states in the valence band, in which the main contribution originated from O 2p orbitals.

## 4. Conclusions

HfO_2_ was found with different polymorphs that confer unique properties to the materials; in the present study, the monoclinic and orthorhombic phases were investigated. In addition, Zr can be used as a dopant agent in different percentages influencing the properties of the systems; thus, it can tune and modulate the devices performances. Although different theoretical and experimental techniques have been already implemented to study the chemical–physical features of these two polymorphs, still a systematic computations investigation of the efficacy of Zr on HfO_2_ material is missing. In this work, the capabilities of the atomistic simulation methods were discussed, shedding light on the possibility to extrapolate different information. With this aim, six different models were generated: for each *P2_1_/c* and *Pca2_1_* polymorph, HfO_2_, Hf_0.75_Zr_0.25_O_2_, and Hf_0.5_Zr_0.5_O_2_ were considered. An extensive comparison of different properties was performed by using the GGA/PBE and GGA/PBE +U methods. The results obtained suggest that the *P2_1_/c* polymorph preserves its properties, highlighting the negligible effect of the Zr defect. Moreover, no internal stress was observed when Zr entities replaced Hf atoms. A different trend was detected for the *Pca2_1_* polymorph, in which an important decrease along the *z*-axis was identified in Hf_0.5_Zr_0.5_O_2_, with important changes in the bond angles. Such a variation stabilized the phase with a significant decrease in terms of internal stress values.

To better describe the electrical and optical properties and the responses to the oxygen vacancies, the GGA + U method was settled and calibrated by using U_Hf_^d^ = 6 eV, U_Zr_^d^ = 5.8 eV, and U_O_^p^ = 4 eV. The PDOS calculation showed that impurity states were located between the CBM and the Fermi level, and this behavior was more evident for o-polymorph. Moreover, following the Mulliken population analysis, the charge-transfer calculations to O atoms indicated completely different results depending on the phases. For the m-phases, Zr doping induced a gradual decrease of charge transfer, while the opposite trend was observed for o-systems. Other important differences were detected by computing the dielectric functions. Minor variations were observed between HfO_2_, Hf_0.75_Zr_0.25_O_2_, and Hf_0.5_Zr_0.5_O_2_ m-polymorphs; on the contrary, distinctive ε_1_ values without applied field were detected for all the o-polymorphs under study, and variations in features for ε_1_ and ε_2_ were identified in the high energy range. Finally, the role of oxygen vacancies on materials’ electrical properties was evaluated, showing a narrowing of the bandgap regardless of the polymorph type. The m-phases showed a higher propensity to suffer oxygen vacancies, and this behavior gradually decreases when the Zr amount has increased. On the other hand, the o-polymorph resulted in being less prone to these defects, and Zr acts as a phase-stabilizing agent and, at the same time, increases the propensity to suffer vacancies.

All of these results provide important information about the physical–chemical properties of Hf_x_Zr_y_O_2_ materials. The combined use of GGA/PBE and GGA/PBE + U methods can provide important guidelines in predicting material properties and guiding the development of HfZrO_2_-based devices.

## Figures and Tables

**Table 1:** Lattice parameters for m- and o-phases of HfO_2_, Hf_0.75_Zr_0.25_O_2_, and Hf_0.5_Zr_0.5_O_2_ according to the GGA–PBE approaches. Lengths (a; b; c) are in Å. Data about monoclinic [20] and orthorhombic [21] HfO_2_ polymorphs are reported for comparison.

Phase	HfO_2_	Hf_0.75_Zr_0.25_ O_2_	Hf_0.5_Zr_0.5_ O_2_	Comparison HfO_2_
m–*P2_1_/c*	a = 5.068	a = 5.065	a = 5.064	a = 5.07
	b = 5.135	b = 5.134	b = 5.135	b = 5.14
	c = 5.292	c = 5.290	c = 5.289	c = 5.29
o–*Pca2_1_*	a = 5.231	a = 5.228	a = 5.229	a = 5.23
	b = 5.008	b = 4.988	b = 5.001	b = 5.00
	c = 5.052	c = 5.052	c = 5.031	c = 5.05

**Table 2:** Average Mulliken population charges.

Model	Species	Total (s + p + d)	Charge (*a.u.*)
*P2_1_/c* HfO_2_	Hf	2.320	1.680
	O	6.840	−0.840
*P2_1_/c* Hf_0.75_Zr_0.25_O_2_	Hf	2.723	1.233
	Zr	2.323	1.023
	O	6.826	−0.826
*P2_1_/c* Hf_0.5_Zr_0.5_O_2_	Hf	3.097	0.987
	Zr	2.309	0.889
	O	6.812	−0.812
*Pca2_1_* HfO_2_	Hf	2.345	1.705
	O	6.852	−0.852
*Pca2_1_* Hf_0.75_Zr_0.25_O_2_	Hf	2.387	1.797
	Zr	2.121	1.531
	O	6.882	−0.882
*Pca2_1_* Hf_0.5_Zr_0.5_O_2_	Hf	2.601	1.931
	Zr	2.365	1.765
	O	6.981	−0.981

**Table 3:** Defect formation energies expressed in eV.

Phase	Hf_x_Zr_y_O_2_ Formation Energy	Hf_x_Zr_y_O_2_–z Formation Energy	Defect Formation Energy
HfO_2_ *P2_1_/c*	−11.32	−11.15	0.17
HfO_2_ *Pca2_1_*	−9.64	−9.27	0.37
Hf_0.75_Zr_0.25_O_2_ *P2_1_/c*	−11.31	−11.13	0.18
Hf_0.75_Zr_0.25_O_2_ *Pca2_1_*	−10.08	−9.59	0.49
Hf_0.5_Zr_0.5_O_2_ *P2_1_/c*	−11.33	−11.12	0.21
Hf_0.5_Zr_0.5_O_2_ *Pca2_1_*	−10.78	−9.96	0.82

## Data Availability

Not applicable.
